# Pseudo-Replication of [GADV]-Proteins and Origin of Life

**DOI:** 10.3390/ijms10041525

**Published:** 2009-04-02

**Authors:** Kenji Ikehara

**Affiliations:** Narasaho College, Rokuyaon-cho 806, Nara, Nara 630-8566, Japan, Fellow of the International Institute for Advanced Studies, Japan, and Emeritus Professor, Department of Chemistry, Nara Women’s University, Japan; E-Mail: ikehara@cc.nara-wu.ac.jp; Tel. +81-742-61-3858; Fax: +81-742-61-8054

**Keywords:** GADV hypothesis, pseudo-replication, [GADV]-protein world, origin of life

## Abstract

The RNA world hypothesis on the origin of life is generally considered as the key to solve the “chicken and egg dilemma” concerning the evolution of genes and proteins as observed in the modern organisms. This hypothesis, however, contains several serious weak points. We have a counterproposal called [GADV]-protein world hypothesis, abbreviated as GADV hypothesis, in which we have suggested that life originated from a [GADV]-protein world, which comprised proteins composed of four amino acids: Gly [G], Ala [A], Asp [D], and Val [V]. A new concept “pseudo-replication” is crucial for the description of the emergence of life. The new hypothesis not only plausibly explains how life originated from the initial chaotic protein world, but also how genes, genetic code, and proteins co-evolved.

## 1. Introduction

**Figure 1.**
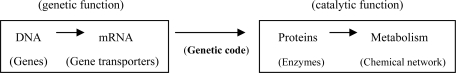
The role of genetic code playing in the fundamental life system of modern organisms.

While genetic information in the form of DNA base sequences or codon sequences is transferred from a parent to progeny cells through DNA replication, the same information is transformed into mRNA and then into amino acid sequence of proteins, according to the genetic code specifications (Figure 1). Organic molecules that the organism needs to live are synthesized by enzyme proteins in metabolic pathways. This process is called metabolism. But, double-stranded DNA, which carries genetic information, cannot be replicated without enzyme proteins, whereas proteins cannot be reproduced without genes. This dilemma made it difficult to account for the origin of life: this is the so-called chicken and egg relationship between genes and proteins in the life system.

**Figure 2.**
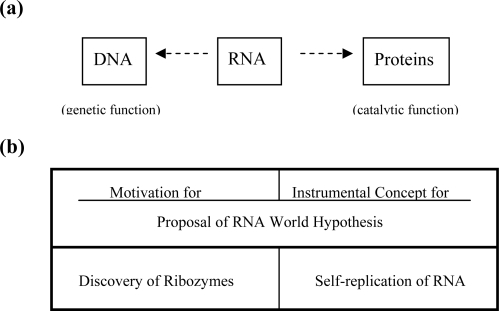
(a) According to the RNA world hypothesis, the genetic function and the catalytic function carried by RNA were transferred to DNA and proteins, respectively. (b) A motivation and an instrumental concept were introduced for proposing the RNA world hypothesis on the origin of life.

However, about 25 years ago, RNA enzymes with some catalytic activities were discovered; they are named ribozymes [1,2]. This discovery suggests that RNA might possess not only genetic function but also catalytic function. Based on this interpretation, Gilbert proposed his RNA world hypothesis of the origin of life [3]. He assumed that RNA had been multiplied and diversified by self-replication to form the RNA world, and that genetic function and catalytic function of RNA were transferred to DNA and proteins, respectively [Figure 2 (a)], resulting in the emergence of life from the RNA world. At the present time, the RNA world hypothesis has widely accepted by many investigators as a key idea for solving the “chicken and egg dilemma” on the origin of life [4]. The discovery of ribozymes gave a motivation for introduction of the new concept or RNA self-replication [Figure 2 (b)].

## 2. Inadequacy of RNA World Hypothesis

In order to explore the origin and evolutionary process of the fundamental life system, we need to understand how genetic function, genetic code, and catalytic function emerged (Figure 1). It has been generally held that acquisition of genetic information must precede the creation of proteins with catalytic functions, because proteins composed of 20 kinds of amino acids are too complex to be produced without the support of genetic encoding. For this reason, the RNA world hypothesis assumed that, at first, genes were selected from a pool of RNA accumulated through RNA self-replication without catalytic proteins, and that proteins were synthesized for catalysis according to the specification of genes. But, genes carrying hereditary information are organized by a linear arrangement of codons and are not formed by polymerization of nucleotides. Rather, the information is specified within each codon by the specific sequence of selected nucleotides [5]. The capability of RNA for self-replication is not relevant to the genetic function for protein synthesis, nor for sequencing codons. This suggests that statistically it is not to be expected to be possible for the first gene to appear encoding a functional water-soluble globular protein in the RNA world, nor create the first genetic code on the primitive Earth.

Furthermore, there are major weak points in the RNA world hypothesis [5,6]: (i) The numbers of atoms (in parentheses) of four nucleotides, AMP (37), UMP (34), GMP (37) or CMP (35), are much larger than those (in parentheses) of four amino acids, Gly (10), Ala (13), Asp (16) or Val (19). This means that the nucleotides are far more complex than the four amino acids. The nucleotides would never be synthesized under pre-biotic conditions through a random combinatory process [4]. Moreover, it must be quite difficult to synthesize even ribose (fructofuranose: a component of nucleotide) having four asymmetric carbon atoms, from simple chemical compounds such as water, carbon dioxide, methane, without proteineous enzymes providing the chiral field for asymmetric synthesis. (ii) Existence of four hydroxyl groups on ribose also makes it difficult to synthesize RNA by joining nucleotides in the absence of enzyme catalysts. In contrast, it is easy to form peptide bond between positive amino group and negative carboxyl group of amino acids. (iii) Self-replication of RNA must be practically impossible due to the following self-contradiction. RNA without any stable tertiary structure would be required to exhibit genetic function as a template, and, simultaneously, RNA would have to be folded into a stable tertiary structure to exhibit its catalytic function [7].

Pre-RNA polymers as genetic materials, which have simpler organic compounds than ribose in their backbones, such as α-threose nucleic acid (TNA) [8–11] and peptide nucleic acid (PNA) [4,12,13], have been proposed to avoid the difficulties of RNA synthesis as described above. Naturally occurring 2’-O-methylated RNA [14] and the split genes [15,16] have also been proposed as an intermediate stage between RNA and DNA and as ancestors of polymeric genes, respectively. A scenario of the origin of life that is not dependent on a dual function of single-stranded RNA has been proposed (the design-by-contract hypothesis) [17]. However, abilities of RNA for protein formation through genetic code are not considered in any modified version of RNA world hypothesis.

We may conclude that it is difficult to explain the emergence of life according to the RNA world hypothesis. We have proposed our GADV hypothesis, suggesting that life originated from [GADV]-protein world, which was formed by pseudo-replication of [GADV]-proteins (see below; G, A, D, and V stand for glycine, alanine, aspartic acid, and valine, respectively).

## 3. GADV Hypothesis about the Origin of Life

### 3.1. The Origin of Genetic Code

Genetic code occupies a core position relating the genetic function to the catalytic function in the life system (Figure 1). We started research exploring the origin of life about 15 years ago: (i) to clarify the origin of the fundamental life system, which involves genes, genetic code and proteins, especially the genetic code. (ii) based thereon, to understand the basic characteristics of the modern genes, the genetic code and proteins.

**Figure 3.**
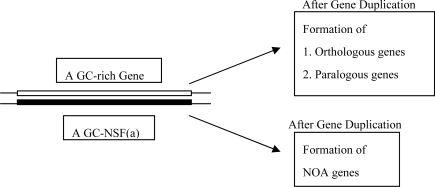
GC-NSF(a) hypothesis for creation of new original ancestor genes (NOA genes), suggesting that NOA genes would be created from non-stop frames on antisense strands of GC-rich genes [GC-NSF(a)] as prescribed by the universal genetic code after gene duplication. On the other hand, homologous genes are created from sense sequences, regardless of GC content.

Here are several main points of our conclusions so far about GADV hypothesis [5,6]. We started from a study on the new original ancestor genes (NOA genes), i.e. the first ancestor genes in gene families consisting of homologous genes. From analyses of microbial genes and proteins obtained from the GenomeNet Database, we found that NOA genes could be produced from non-stop frames on anti-sense strands of, not AT-rich, but GC-rich microbial genes [GC-NSF(a)] [18] (Figure 3). This conclusion was mainly based on the facts that hypothetical proteins encoded by GC-NSF(a)s satisfied six conditions for folding of polypeptide chains into water-soluble globular proteins (hydropathy, α-helix, β-sheet and turn/coil structure formations, acidic amino acid and basic amino acid compositions) and that the probability of stop codon appearance is sufficiently small to produce non-stop frames on the GC-NSF(a)s [18]. The six conditions were obtained by examining if each of six values fell into the interval between the average values of extant proteins plus/minus standard deviations. Those average values of most proteins held nearly-constant levels, regardless of GC contents, which were obtained by calculation using amino acid structural indexes [19] and amino acid compositions of currently observed microbial proteins encoded by seven microbial genomes with different GC contents [5,20]. One reason why GC-NSF(a)s well satisfy the six conditions is that base compositions at three codon positions on sense and antisense strands are rather similar [5,18]. We also found that the base composition format of highly GC-rich genes (65~75%) and hypothetical sequences of GC-NSF(a) are approximate repetitions of SNS, where S means G or C. This result suggests that the sequences of SNS repetitions might hold a strong potential to function as genes. Further, we looked for a minimum set of amino acids that could produce proteins satisfying four conditions (hydropacy, capabilities of forming α-helix, β-sheet, and turn/coil). It was found that [GADV]-proteins encoded by GNC code satisfied the four conditions, when about equal amounts of [GADV]-amino acids were contained in the proteins [20], but all four amino acids encoded by other codons in rows and columns in the universal genetic code table did not satisfy at least one of the four conditions, except for the GNG code, a slightly modified form of the GNC code, where N means either of four nucleobases (G, C, A, and T or U). The results of this search indicate that a group of four amino acids (G, A, D and V) could produce proteins that are basically comparable in their potential to produce contemporary proteins forming secondary or tertiary structures.

**Figure 4.**
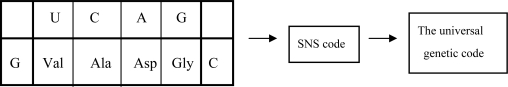
GNC-SNS hypothesis on the origin and evolutionary pathway of the genetic code, suggesting that the universal genetic code originated from GNC primeval genetic code through SNS primitive genetic code.

We noticed that GNC-SNS primitive genetic code hypothesis [20] implied that universal genetic code (NNN: 4× 4× 4 = 4^3^ = 64 codons) was accounted for as follows: the universal code is represented formally and substantially by triplets. But, it originated from GNC code, which was formally represented by triplets but substantially implemented as singlets. The GNC code comprises four codons (1× 4× 1 = 4^1^ = 4) and four [GADV]-amino acids. It could derive through formally triplet and substantially doublet SNS code, consisting of 16 codons (2× 4× 2 = 4^2^ = 16) and 10 amino acids ([GADV]-amino acids plus Glu, Leu, Pro, His, Gln and Arg) (Figure 4).

[GADV]-amino acids and SNS encoding amino acids are located in one row and in four rows of the genetic code table, respectively. This indicates that the genetic code table not only represents a relationship between codons and amino acids, but also reflects a framework for creation of NOA proteins.

It is well known that serine, α-aminobutyric acid, and α-aminoisobutyric acid, can be synthesized from simple chemical compounds under pre-biotic conditions [21] and in extraterrestrial environments as evidenced by meteorites [22]. But, those amino acids were not used in the most primitive genetic code of the four amino acids (Figure 4), for the following reasons. Serine is a small hydrophilic amino acid with high turn/coil formability like glycine, while both α-aminobutyric acid and alanine, with non-branched side chain, are α-helix forming amino acids. Glycine and alanine with simpler structure than serine and α-aminobutyric acid would be selected for the most primitive code, respectively. The α-aminoisobutyric acid is an achiral amino acid with two methyl groups attached to an α-carbon atom. We assumed that this amino acid was not used in addition to another achiral glycine in the most primitive code for regular structure formation due to the large turn/coil formability.

### 3.2. Pseudo-replication of [GADV]-Proteins in the Absence of Genetic Function

Discussion on protein structure formation usually begins with the primary structure or amino acid sequence of the protein, not with amino acid composition. Although we happened to use amino acid composition for investigation of protein structure formability, it resulted in interesting conclusions, as described above.

Structure formability is the same for any protein of the same amino acid composition, that was randomly selected for assembling. This means that every protein synthesized by random peptide bond formation among amino acids in the amino acid composition could be folded into similar but into different structures. Proteins can have the same amino acid composition but different sequences. We call such a specific amino acid composition that is favorable for protein structure formation “protein 0^th^-order structure” [5].

The notion of the protein 0^th^-order structure led us to a new concept, assuming that water-soluble globular [GADV]-proteins could be created by random polymerization of [GADV]-amino acids with a high probability, even in the absence of any genetic function, i.e., before the creation of the first gene. This is because individual [GADV]-amino acids are functional units for protein structure formation, and [GADV]-amino acids satisfy the four conditions for formation of water-soluble globular proteins [20]. Previous experimental results showed that [GADV]-peptides have protease activities, implying that [GADV]-peptides carry catalytic activity for peptide bond formation through micro-reversibility of catalysts [23]. This suggests that [GADV]-proteins could be pseudo-replicated even before creation of the first (GNC)_n_ gene, due to the simple amino acid composition and high activities catalyzing peptide bond formation of the proteins. Pseudo-replication is a process where proteins comprising the same constituent set of amino acids (composition), which possess similar but different structures, are generated by a random process without resorting to any exact duplication.

**Figure 5.**
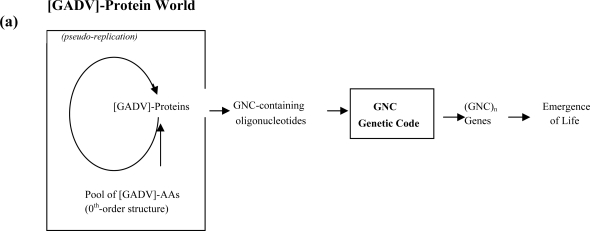

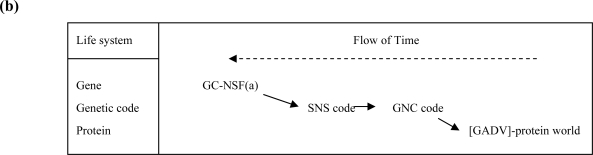
(a) [GADV]-protein world hypothesis (GADV hypothesis) about the origin of life: Life originated from the [GADV]-protein world, which was created by pseudo-replication of [GADV]-proteins i.e., random polymerization of [GADV]-amino acids in a specific amino acid composition (a protein 0^th^-order structure). Life emerged from the [GADV]-protein world through generating GNC primeval genetic code. (b) Development of [GADV]-protein world hypothesis on the origin of life. Solid lines and a dotted line show the history and time flow, respectively.

Various water-soluble globular [GADV]-proteins, carrying quite different amino acid sequences to be represented by different structures and serving different functions, can be produced by the pseudo-replication. The notion of the random polymerization of [GADV]-amino acids led us to the new scenario about the origin of life: the GADV hypothesis. Life emerged from [GADV]-protein world [Figure 5 (a)] [5,6]. The development of the GADV hypothesis based on the GC-NSF(a) hypothesis is summarized in Figure 5 (b).

### 3.3. Emergence of Life from [GADV]-Protein World

**Figure 6.**
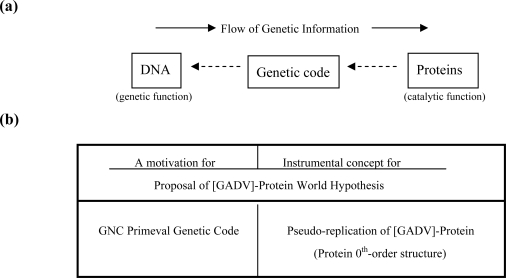
(a) Formation processes of the life system (chicken and egg relationship between DNA and proteins) viewed from the GADV hypothesis. (b) The motivation and the new instrumental concept for the GADV hypothesis on the origin of life.

A possible evolutionary process of emergence of life based upon the GADV hypothesis is as follows [5,6,23]. [GADV]-amino acids were synthesized on the primitive Earth. It is well known that [GADV]-amino acids can be easily synthesized in Miller type experiments [24–28]. [GADV]-proteins were produced, for example, by repeated heat-drying processes of [GADV]-amino acids in tide pools on the primitive Earth, and were further accumulated by pseudo-replication to form [GADV]-protein world. Subsequently, nucleotides and oligonucleotides were synthesized by their high catalytic activities in the world. The accumulation of oligonucleotides triggered the generation of GNC primeval genetic code through stereospecific complex formation among four [GADV]-amino acids and four corresponding GNC-containing oligonucletoides [29,30] [Figures 5 (a) and 6 (a)]. More efficient synthesis of [GADV]-proteins with the complexes than direct synthesis among individual [GADV]-amino acids assisted establishing the GNC primeval genetic code generation.

Next, GNC-repeating sequences were produced by random phosphodiester bond formation on chiral [GADV]-proteins or by linear arrangement of GNC codons in the complexes of GNC-containing oligonucleotides and [GADV]-amino acids. Thus, the first single-stranded (GNC)_n_ gene was created, when one (GNC)_n_ sequence encoding a [GADV]-protein with the required function was selected from a pool of (GNC)_n_ polynucleotides, leading to the emergence of the first life [Figure 5 (a)]. How the “chicken and egg relationship” between genes and proteins was formed on the primitive Earth also can be explained from the standpoint of GADV hypothesis as going up from the lower ([GADV]-protein synthesis) to the upper stream (creation of genes) of the genetic flow [Figure 6 (a)]. In the RNA world hypothesis, it seems difficult to find a reasonable strategy for creation of the first gene. The notion of GNC primeval genetic code gave a motivation for introduction of the new concept or pseudo-replication of [GADV]-proteins [Figure 6 (b)].

## 4. Justification of GADV Hypothesis about the Origin of Life

GADV hypothesis is consistent with three general principles for the emergence of significant organization.

### 4.1. General Principle 1: From Simple to Complex Molecules

Ordinary, simpler organic molecules should be formed earlier than more complex molecules, in an era of chemical evolution, since the number of combinations for synthesis of organic compounds becomes larger not additively but exponentially, as the number of atoms in the molecule becomes larger. Therefore, it is natural to assume that accumulation of [GADV]-amino acids and [GADV]-proteins on the primitive Earth preceded the appearance of nucleotides and RNA.

### 4.2. General Principle 2: From Random to Well-organized Processes

Every event for creating a system must start as a random process. In the GADV hypothesis, the first functional [GADV]-protein was produced by random peptide bond formation in the pool of [GADV]-amino acids before the first (GNC)_n_ gene appeared. Subsequently, the most primeval GNC genetic code and the first (GNC)_n_ gene appeared and reproduced useful [GADV]-proteins that were needed to develop the system.

### 4.3. General principle 3: From Catalytic to Genetic Functions

Genes are organized as codon sequences and cannot be created by random polymerization of nucleotides. A genetic system, for this reason, cannot precede the appearance of catalytic enzymes: A gene cannot be generated prior to the crucial protein (enzyme) synthesis. In contrast, appearance of [GADV]-proteins with catalytic functions preceded the appearance of the genetic system.

## 5. Production of NOA Proteins after Creation of Double-stranded Gene

### 5.1. Mechanisms for Creation of New Genes and New Proteins

**Figure 7.**
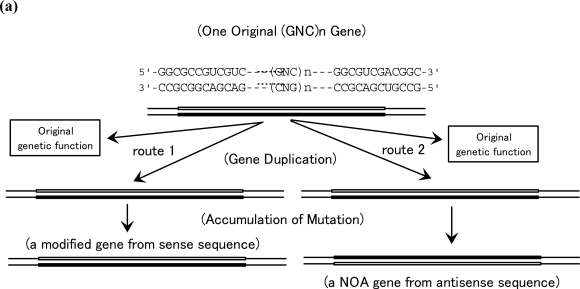

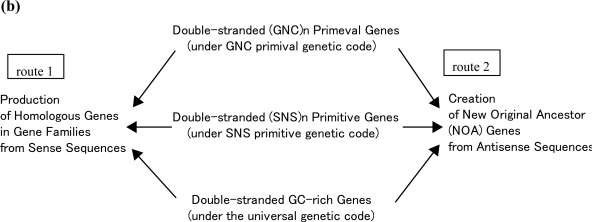
Two routes for creation of new genes. (a) While new (GNC)_n_ genes homologous with parental gene were produced from GNC codon sequences on the sense strand (route 1), NOA genes were created from GNC codon sequences on the anti-sense strands (route 2). (b) Two routes 1 and 2 similar to those as shown in (a) would be utilized for creation of new genes, when necessary, always after creation of the first double-stranded (GNC)_n_ gene.

The first gene must have been a single-stranded (GNC)_n_ gene, since genetic information must be composed of only GNC codons under GNC primeval genetic code. Subsequently, the first double-stranded (GNC)_n_ gene was produced by synthesis of complementary strand of a single-stranded (GNC)_n_ gene. Of course, base compositions at three positions within each codon on the sense strands are similar to those on the corresponding anti-sense strands. Presumably, GNC codon sequences on anti-sense strands were utilized as genes encoding NOA proteins, which were the first proteins in protein families. GNC codon sequences on antisense strands are quite different from sense sequences so that can be regarded as random arrangement of GNC codons [31] [Figure 7 (a)]. This implies that sense sequences were utilized for encoding not the same but similar information for homologous proteins in a protein family, as proposed by Ohno [route 1, Figure 7 (a)] [32], while anti-sense sequences produced as part of the consequence of gene duplication gave an opportunity for creation of NOA proteins that are quite different from all existing proteins [route 2, Figure 7 (a)] [18].

(SNS)_n_ genes were utilized for synthesizing proteins composed of 10 amino acids encoded by SNS code, after evolution of SNS primitive genetic code from GNC primeval genetic code. In the era of double-stranded (SNS)_n_ genes, SNS codon sequences on anti-sense strands were utilized for creating NOA genes, encoding NOA proteins, given that those are actually regarded as random arrangement of SNS codons. This situation is similar to the case of (GNC)_n_ genes described above [Figure 7 (a)].

Even after the formation of the universal genetic code using (NNN)_n_ as genetically meaningful sequences, new genes were always created by utilization of sense and anti-sense sequences of the previously existing genes, not by direct polymerization of nucleotides or even by random joining of triplet base sequences or codons. Actually, codon sequences on GC-NSF(a)s are utilized for synthesis of NOA proteins, since the sequences are fairly similar to (SNS)_n_ sequences, which may be regarded as relics of the (SNS)_n_ sequences [5,18,20,31].

Thus, NOA proteins were created in effect, though not actually, by random polymerization of amino acids in specific amino acid compositions, which are in accordance to the codon sequences on the anti-sense strands of (GNC)_n_ (encoding [GADV]-amino acids) and (SNS)_n_ (encoding ten amino acids ([GADV]-amino acids plus Glu [E], Leu [L], Pro [P], His [H], Gln [Q], and Arg [R])), and of GC-rich genes [Figure 7 (b)].

### 5.2. Group Coding under GNC and SNS Codes

GNC or SNS code constitutes a part of the universal genetic code or NNN code. Therefore, under the GNC or SNS code, group coding which imposes constraints on genetic information to be GNC or SNS codon sequences, for production of functional proteins, should be adopted to avoid meeting stop codons. Otherwise, non-assigned triplets that serve stop codons, would appear at an extremely high frequency. Presumably, sufficiently reliable replication of the (GNC)_n_ genes and of (SNS)_n_ sequences enabled the group coding. Base sequences were abandoned as inactive genes, when codons other than GNC and SNS appeared on the sequences.

## 6. Conclusions

The RNA world hypothesis is widely accepted at present, but this hypothesis cannot explain how the first gene and the most primitive genetic code emerged on the primitive Earth. In contrast, GADV hypothesis can plausibly explain not only how the most primitive genetic code was introduced on the primitive Earth and how the first gene encoding the first protein was created, but also it suggests how the “chicken and egg relationship” observed between gene and protein took place.

**Figure 8.**
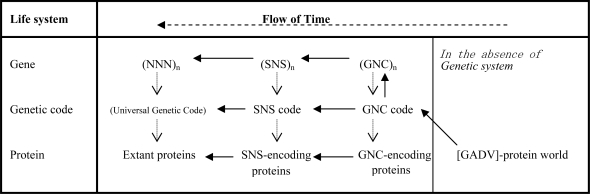
A possible evolutionary pathway of the life system, which comprised genes, genetic code (codons for amino acids), and proteins, and originated from the [GADV]-protein world. Solid arrows, dotted arrows and a broken arrow indicate directions of evolutionary pathways of the life system, the expression of genetic information, and the time flow, respectively.

According to the GADV hypothesis, the origin and evolutionary processes of the life system are described (Figure 8): (i) [GADV]-protein world was created by pseudo-replication of [GADV]-proteins, which were generated by random polymerization of [GADV]-amino acids in the absence of any gene. Formation of the [GADV]-protein world by pseudo-replication of [GADV]-proteins introduced the first life on the primitive Earth; (ii) the GNC primeval genetic code was created through a specific interaction between [GADV]-amino acids and GNC-containing oligonucleotides; (iii) the most primitive single-stranded (GNC)_n_ gene was created by random concatenation of GNC; (iv) after the emergence of the first double-stranded (GNC)_n_ gene, [GADV]-proteins were produced, according to the codes of diverse (GNC)_n_ genes created from both sense and anti-sense codon sequences; (v) genes, genetic code, and proteins co-evolved from (GNC)_n_ primeval genes, GNC primeval genetic code, and [GADV]-proteins, respectively. As the results, the modern life system was created comprising (NNN)_n_ genes, the universal genetic code, and modern proteins, transformed through primitive (SNS)_n_ genes, SNS code, and SNS-encoding proteins, respectively.

In providing plausible accounts about the origin and evolutionary processes of the fundamental life system, GADV hypothesis conforms to generally acceptable principles of evolution: (i) from simple to complex molecules; (ii) from random to well-organized processes; (iii) from catalytic to genetic functions. Thus we may speculate with some confidence that life evolved from the [GADV]-protein world into an RNA-protein world. The RNA world was never formed through RNA self-replication with RNA catalysts.
